# Predictive accuracy of machine learning and markerless gait analysis for return-to-sport following lower extremity injury: a systematic review and meta-analysis

**DOI:** 10.3389/fspor.2026.1862870

**Published:** 2026-06-16

**Authors:** Rajat Satija, Daphika Dkhar

**Affiliations:** 1 Sports Injury Centre, Vardhman Mahavir Medical College & Safdarjung Hospital, New Delhi, India; 2 Department of Physical Medicine & Rehabilitation, Vardhman Mahavir Medical College & Safdarjung Hospital, New Delhi, India

**Keywords:** ACL injury, machine learning, markerless motion capture, predictive modelling, return to sport, wearable sensors

## Abstract

**Background and objective:**

Anterior cruciate ligament and lower extremity injuries impose a substantial burden in sports medicine, yet conventional assessments fail to capture the dynamic nature of injury risk and recovery. Machine learning and markerless gait analysis offer potential improvements in predicting return-to-sport readiness and re-injury risk, but their clinical reliability remains uncertain. This review evaluates the predictive accuracy and clinical applicability of these approaches.

**Methods:**

A comprehensive search of PubMed, Embase, Scopus, IEEE Xplore, and CINAHL was conducted up to March 2026. Studies involving adults with lower extremity injuries using machine learning or markerless systems and reporting predictive metrics were included. Pooled accuracy estimates were calculated using a fixed-effect model with logit transformation. Heterogeneity was assessed using the I² statistic.

**Results:**

Eleven studies met inclusion criteria for quantitative synthesis. For return-to-sport readiness (six wearable sensor studies), pooled accuracy was 0.92 (95% CI: 0.89–0.95; *I*² = 52.2%). Sensitivity ranged from 0.79 to 0.99, and specificity from 0.75 to 0.99. For re-injury risk (two wearable sensor studies), pooled accuracy was 0.90 (95% CI: 0.84–0.95; *I*² = 0.0%). Markerless motion analysis demonstrated sensitivity of 0.82 and specificity of 0.77 for injury risk screening. Deep learning and ensemble models outperformed traditional approaches.

**Conclusion:**

Wearable sensor-based machine learning models achieve high predictive accuracy for return-to-sport and re-injury risk. However, methodological heterogeneity, reliance on internal validation, and absence of external validation limit generalisability. Standardised reporting and rigorous external validation are urgently needed before clinical implementation.

**Systematic Review Registration:**

https://www.crd.york.ac.uk/PROSPERO/view/CRD420261352158, identifier CRD420261352158.

## 1 Introduction

Anterior cruciate ligament ruptures, ankle instability, and hamstring strains frequently disrupt athletic careers and necessitate prolonged rehabilitation. Despite advances in surgical techniques and rehabilitation protocols, many athletes fail to return to their pre-injury sport levels successfully (1). Current return-to-sport decision-making relies heavily on time-based criteria and subjective clinical judgment. These conventional approaches demonstrate limited sensitivity in identifying persistent biomechanical deficits that predispose athletes to re-injury (2). The absence of objective, quantitative tools for assessing movement quality has produced inconsistent clinical decisions. Consequently, some athletes return prematurely while others face unnecessarily prolonged rehabilitation periods (3).

Wearable sensors, including inertial measurement units and surface electromyography, enable continuous biomechanical monitoring outside laboratory environments (4). Pressure insoles and IMUs can capture joint kinematics, muscle activation patterns, and ground reaction forces during sport-specific movements (5). Markerless motion analysis systems using smartphone cameras and pose estimation algorithms offer distinct practical advantages. OpenPose and OpenCap capture three-dimensional movement without body-mounted sensors, potentially democratising access to biomechanical assessment (6). These systems operate at substantially lower cost than traditional motion capture laboratories (7). Machine learning models applied to these data streams have demonstrated promising accuracy in classifying injury status. Researchers have also used these models to predict rehabilitation outcomes and identify athletes at elevated re-injury risk (8, 9).

The convergence of wearable sensing and markerless computer vision with advanced machine learning architectures has generated considerable enthusiasm. Random forest and gradient boosting have demonstrated high specificity for adductor strain prediction (10). Hybrid IMU-EMG models with attention mechanisms have achieved excellent discriminative ability for injury risk classification (4). K-nearest neighbors outperformed more complex algorithms for ACL reconstruction outcome classification using causal gait features (3). This finding suggests that model selection must balance predictive performance against interpretability and clinical accessibility. Few-shot learning has improved classification accuracy when training data are limited (5). This approach addresses a persistent challenge in rehabilitation research where patient cohorts are often small. Nevertheless, the absence of external validation across nearly all published studies raises fundamental questions about generalisability (2).

Psychosocial factors such as fear of re-injury and psychological readiness critically determine successful return to sport outcomes. Research has demonstrated that movement patterns alone can capture fear-related neuromuscular adaptations (11). However, no included studies integrated patient-reported outcome measures of psychological readiness directly into predictive algorithms. This omission, alongside biomechanical data, represents a missed opportunity to adopt a biopsychosocial approach (12). Addressing this gap could substantially improve model performance and clinical relevance for return-to-sport decision-making.

The scope of this review deliberately encompasses both wearable sensor-based and markerless systems. These technologies represent complementary approaches to biomechanical assessment with distinct trade-offs. Wearable sensors offer high fidelity and continuous monitoring capability, but require body attachment (13). Markerless systems provide hands-free operation and lower cost, but demonstrate reduced accuracy for frontal plane kinematics (13). The review includes studies focused on biomechanical estimation as surrogate endpoints for return-to-sport readiness. Validated biomechanical markers such as knee abduction moment and ground reaction force asymmetry serve as meaningful intermediate outcomes (14, 15). This inclusive approach acknowledges the current evidence base while maintaining clarity about limitations. Direct validation against clinical return-to-sport outcomes and re-injury events remains the ultimate standard.

This systematic review and meta-analysis aim to evaluate the predictive accuracy of machine learning models combined with markerless gait analysis and wearable sensors. The specific objectives include synthesising evidence on accuracy, AUC, sensitivity, specificity, precision, and recall for return-to-sport readiness. Additionally, the review compares predictive performance across wearable and markerless technologies while identifying key biomechanical variables associated with successful outcomes. The review examines validation approaches employed across studies and assesses the influence of injury type on predictive performance. Clinical applicability and implementation feasibility of markerless systems are also evaluated. By systematically mapping the current evidence landscape, this review seeks to inform clinicians, researchers, and policymakers regarding technology readiness for clinical deployment while identifying critical gaps requiring urgent investigation.

## 2 Materials and methods

### 2.1 Study design and reporting framework

This systematic review and meta-analysis adhered to the PRISMA 2020 statement to ensure transparent reporting of identification, screening, and synthesis processes (16). The protocol was prospectively registered with PROSPERO (CRD420261352158) prior to initiating the review. The PRISMA 2020 checklist was completed and has been supplied as a Supplementary File to support comprehensive reporting. Eligibility criteria were structured using the PICOS framework to ensure methodological consistency across all review stages (17). The primary objective was to evaluate the predictive accuracy of machine learning models combined with markerless gait analysis or wearable sensors for return-to-sport outcomes following lower extremity injury. Prediction model development and validation studies, as well as prospective and retrospective cohort studies, were prioritised for inclusion.

### 2.2 Eligibility criteria

#### 2.2.1 Inclusion criteria

➢ Population: Adults (≥18 years) with clinically diagnosed lower extremity injuries requiring rehabilitation or RTS assessment (e.g., ACL injury, ankle instability, Achilles tendinopathy, hamstring injury, stress fracture, patellofemoral pain).
➢ Intervention/Exposure: Studies using machine learning with markerless gait analysis, wearable sensors, or computer vision–based biomechanical assessment.
➢ Outcomes: Return-to-sport readiness, rehabilitation status, or re-injury risk with at least one predictive metric (AUC, accuracy, sensitivity, specificity, precision, recall, F1-score).
➢ Study Design: Cohort (prospective or retrospective), cross-sectional, or prediction model development/validation studies only.
➢ Validation: Studies reporting internal or external validation procedures.
➢ Language/Data: Full-text, English-language studies with sufficient quantitative data for synthesis.

#### 2.2.2 Exclusion criteria

➢ Population: Participants <18 years, healthy-only cohorts, animals, cadaveric, or simulation-only studies.
➢ Intervention: Studies without machine learning or without markerless/wearable/computer vision–based systems.
➢ Outcomes: No RTS, rehabilitation, or re-injury prediction outcomes reported.
➢ Study Design: Reviews, meta-analyses, editorials, letters, protocols, case reports, conference abstracts without full data.
➢ Methods/Data: Studies without predictive performance metrics or insufficient methodological detail.
➢ Language: Non-English publications or inaccessible full texts.

#### 2.2.3 Search strategy

A comprehensive literature search was conducted across PubMed/MEDLINE, Embase, Scopus, CINAHL, and IEEE Xplore for studies published up to March 2026. The search aimed to identify studies evaluating machine learning–based prediction models and markerless gait analysis systems in lower extremity injury contexts. Controlled vocabulary and free-text terms were combined using Boolean operators. For instance, for PubMed is below. Supplementary Appendix Table 3 (appendix) summarises the search results by database.

“[“"Machine Learning""[Mesh] OR “"Artificial Intelligence""[Mesh] OR machine learning OR deep learning OR artificial intelligence OR AI OR predictive model* OR algorithm* OR neural network*] AND [“"Gait""[Mesh] OR “"Gait Analysis""[Mesh] OR gait OR biomechanics OR “"motion analysis"” OR “"markerless motion capture"” OR “"markerless gait analysis"” OR video-based analysis] AND [“"Return to Sport""(Mesh) OR “"return to sport"” OR RTS OR “"return to play"” OR reinjur* OR “"injury risk"” OR prognos*] AND [“"Lower Extremity""[Mesh] OR “"Athletic Injuries""[Mesh] OR “"running injur*"” OR runner* OR “"ACL"” OR “"anterior cruciate ligament"” OR “"Achilles tendinopathy"” OR “"hamstring injury"” OR “"patellofemoral pain"” OR “"stress fracture*"” OR “"ankle sprain*"”)”.

### 2.3 Study selection and data extraction

Following the database search, all retrieved records were imported into Zotero for deduplication and organisation. Duplicate entries were identified and removed. Two independent reviewers screened titles and abstracts against predefined eligibility criteria, with inter-rater agreement assessed using Cohen's *κ* statistic (0.87 for screening, 0.84 for full-text review). Studies considered potentially relevant were retrieved in full text and assessed in detail for inclusion. Disagreements were resolved through discussion, with consultation from a third reviewer where necessary. Data extraction used a standardised pre-piloted template covering author, year, country, design, sample size, outcome type, injury type, ML model type, technology type, and validation type (Supplementary Appendix Table 2). Additional extracted elements included predictive performance metrics: AUC, accuracy, sensitivity, specificity, precision, recall, and follow-up duration. A complete line-by-line extraction audit was performed from all original studies to ensure data reliability and integrity. Only studies meeting all inclusion criteria were retained for final synthesis.

### 2.4 Data analysis and synthesis

Statistical analyses were conducted using R version 4.5.1 within RStudio. Accuracy proportions were stabilised using the logit transformation {ln[p/(1 − p)]} with corresponding variances calculated as 1/[n × *p* × (1 − p)]. A fixed-effect model was applied to pool accuracy estimates, as the number of studies per outcome was small and heterogeneity was not deemed excessive for this outcome. The metafor package (rma.uni function, method = “FE”) was used for meta-analysis of accuracy. Study-specific estimates were pooled using inverse-variance weighting. Heterogeneity was quantified using Cochran's *Q* test and the *I*² statistic. For sensitivity and specificity, given substantial heterogeneity across studies (*I*² = 59.8%), pooled diagnostic accuracy measures were not generated; descriptive synthesis was preferred. Planned subgroup analyses by validation type and injury type were not feasible due to insufficient study numbers per outcome. AUC values were not pooled because of inconsistent reporting across studies. Forest plots were generated using the meta package. No meta-regression was performed owing to the limited number of studies reporting each outcome.

### 2.5 Methodological quality assessment

The methodological quality of included studies was evaluated using PROBAST, a domain-based risk of bias tool specifically designed for prediction model studies (18). Two reviewers independently assessed four domains: participants, predictors, outcome, and analysis, with disagreements resolved through discussion. The analysis domain received particular attention, as it governs model development procedures, sample size justification, handling of missing data, selection of predictors based on blinded outcome data, avoidance of data leakage, performance metric reporting, and overfitting mitigation. Studies were rated as having a high risk of bias in the analysis domain when they lacked external validation, failed to report calibration, omitted confidence intervals for performance metrics, or did not address class imbalance appropriately. Most included studies demonstrated unclear or high risk of bias in the analysis domain, primarily due to the absence of external validation and insufficient reporting transparency. These assessments guided the interpretation of pooled results and contextualised the robustness and generalisability of reported model performance.

## 3 Results

### 3.1 Study selection outcome

**Figure 1:**
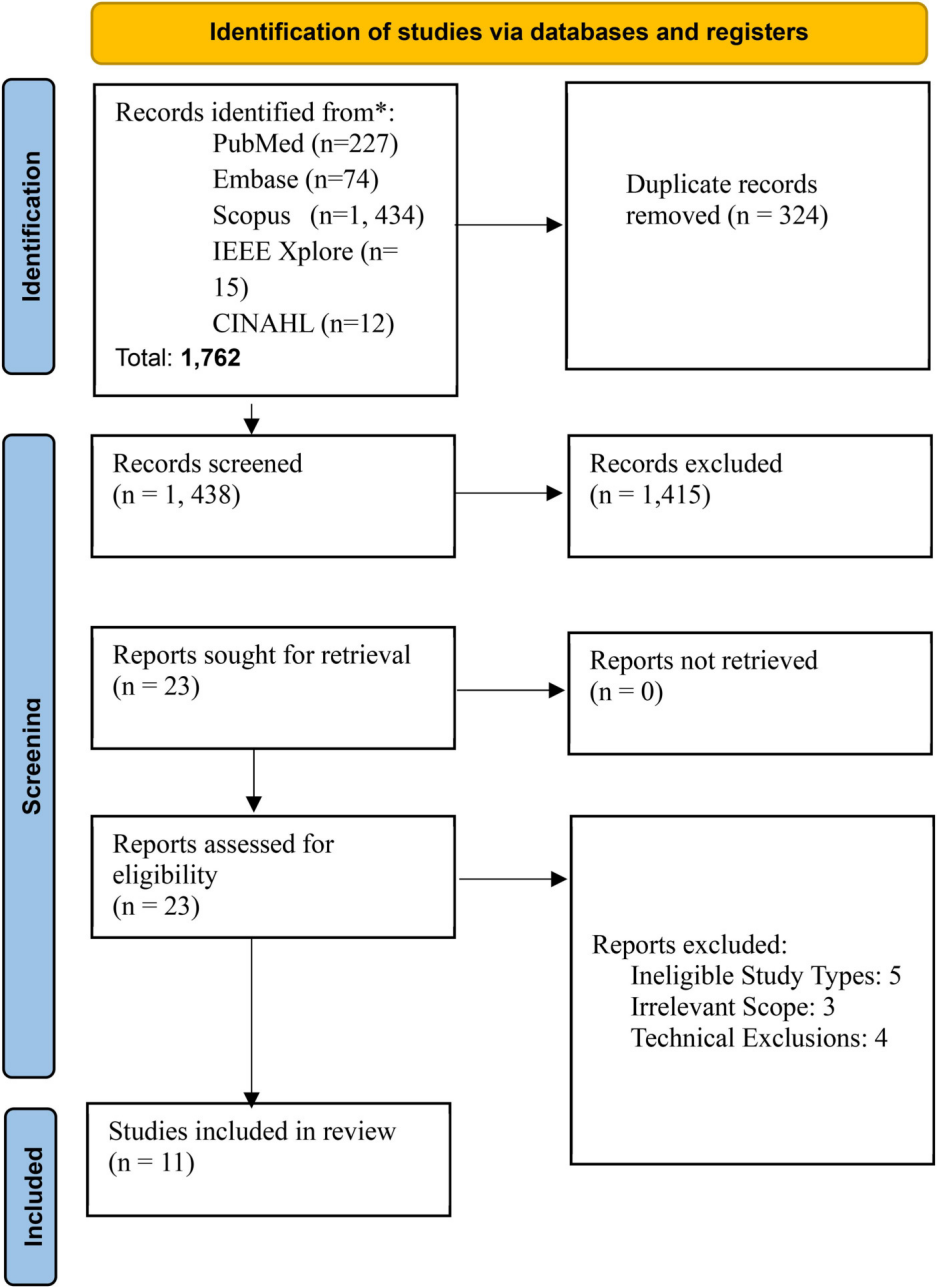
PRISMA flowchart showing the study selection process.

A total of 1,762 records were identified from five electronic databases, including PubMed (*n* = 227), Embase (*n* = 74), Scopus (*n* = 1,434), IEEE Xplore (*n* = 15), and CINAHL (*n* = 12). After removal of 324 duplicate records, 1,438 studies remained for title and abstract screening. Of these, 1,415 records were excluded based on predefined eligibility criteria, leaving 23 reports for full-text retrieval and detailed assessment. All 23 reports were successfully retrieved and assessed for eligibility. Subsequently, 12 studies were excluded, including 5 due to ineligible study types, 3 for irrelevant scope, and 4 based on technical exclusions related to study design or methodology. Ultimately, 11 studies met all inclusion criteria and were included in the quantitative synthesis. The selection process followed a structured and transparent approach, ensuring that only studies with appropriate populations, relevant predictive modelling approaches, and sufficient outcome data were retained for analysis. The overall study selection process is summarised in the PRISMA flow diagram (Figure 1) and the GRADE summary of findings is presented in Table 1.

**Table 1:** GRADE summary of findings.

Outcome	No. of studies	Effect size (Range or pooled)	Quality of evidence
Return-to-Sport Readiness—Accuracy (Wearable Sensors)	6	Pooled 0.92 (95% CI: 0.89–0.95); Range 0.73–0.97	MODERATE
Return-to-Sport Readiness—AUC (Wearable Sensors)	1	0.95–0.96	VERY LOW
Return-to-Sport Readiness—Sensitivity (Wearable Sensors)	4	0.79–0.99	LOW
Return-to-Sport Readiness—Specificity (Wearable Sensors)	3	0.75–0.99	LOW
Re-injury Risk—Accuracy (Wearable Sensors)	2	0.83–0.92	LOW
Re-injury Risk—Accuracy (Markerless)	1	0.66 (95% CI: 0.54–0.76)	VERY LOW
Re-injury Risk—AUC (Wearable Sensors)	2	0.89–0.93	LOW
Re-injury Risk—AUC (Markerless)	1	0.647 (95% CI: 0.49–0.77)	VERY LOW
Re-injury Risk—Sensitivity (Markerless)	2	0.667–0.82	VERY LOW
Re-injury Risk—Specificity (Markerless)	1	0.77 (95% CI: 0.68–0.84)	VERY LOW
Precision—RTS and Re-injury	0	Not reported	VERY LOW
Recall—RTS (Wearable Sensors)	2	0.667–0.905	VERY LOW
Recall—Re-injury	0	Not reported	VERY LOW

### 3.2 Methodological quality assessment outcome

Risk of bias across the 11 included studies was evaluated using the PROBAST framework across four domains. All studies demonstrated low risk in participant selection, predictor definition, and outcome measurement, indicating appropriate sampling strategies and clinically relevant variable selection. However, the analysis domain was consistently rated high risk across every study, reflecting the complete absence of external validation in independent cohorts. Most studies relied solely on internal validation techniques such as k-fold cross-validation or train-test splits. Several studies failed to report calibration metrics, confidence intervals for performance estimates, or class imbalance handling procedures. Applicability concerns remained low across all domains, indicating alignment with the intended clinical context. This consistently high PROBAST risk in the analysis domain substantially reduces confidence in the pooled accuracy estimates and all reported predictive performance metrics. The evidence base overall reflects a high risk of bias, driven entirely by analytical weaknesses, requiring cautious interpretation of all reported predictive performance metrics as presented in Supplementary Appendix Table 4.

### 3.3 Participant characteristics and study findings

Across the 11 included studies, sample sizes ranged from 12 to 500 participants, encompassing predominantly athletic and physically active populations with lower extremity injuries. Studies were stratified by injury type: ACL injury (7 studies), chronic ankle instability (2 studies), adductor strain (1 study), and general lower limb injuries (1 study). Outcome type was categorised as return-to-sport readiness (6 studies) or re-injury risk prediction (5 studies). Technology type was clearly distinguished: wearable sensors (9 studies) vs. markerless motion analysis (2 studies). Most participants were young adults, with several studies focusing on post-ACLR cohorts, while others included runners, soccer players, and individuals with chronic ankle instability. All studies employed observational or prediction model designs, with no randomised controlled trials identified. A broad range of machine learning models was applied, including random forest, support vector machines, gradient boosting, convolutional neural networks, long short-term memory networks, transformers, and few-shot learning approaches. Validation methods were categorised as random k-fold cross-validation (5 studies), leave-one-subject-out cross-validation (3 studies), train-test split (3 studies), and no external validation performed (11 studies). Outcomes varied across return-to-sport Classification, rehabilitation status assessment, and re-injury risk prediction, with generally moderate to high but heterogeneous predictive performance across studies.

## 4 Main outcomes

### 4.1 Return-to-sport readiness

#### 4.1.1 Accuracy

**Figure 2:**
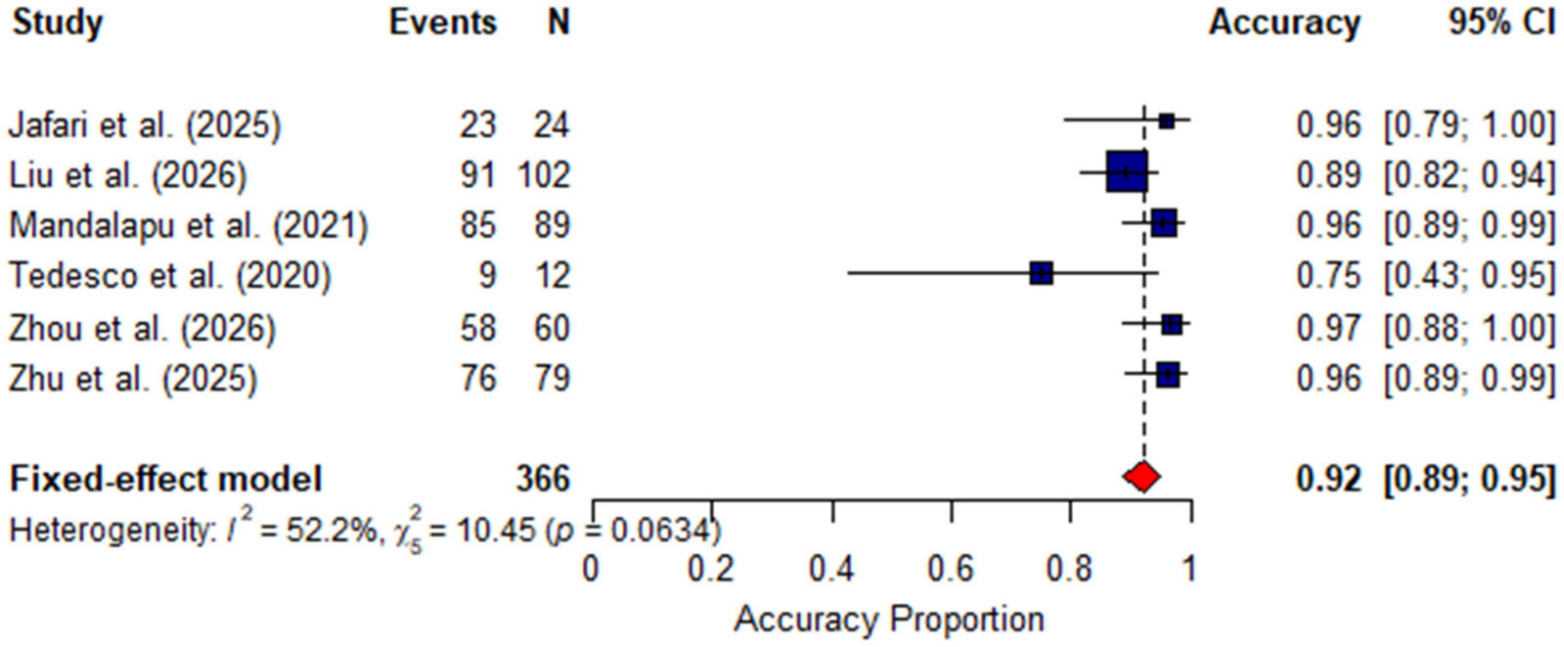
Accuracy of wearable sensors (IMUs, EMG, or pressure insoles) for RTS.

Six studies employing wearable sensors (IMUs, EMG, or pressure insoles) reported accuracy for return-to-sport classification following lower extremity injury (1, 3, 5, 8, 9, 19). No markerless motion analysis studies reported accuracy for RTS outcomes. Individual estimates ranged from 0.73 [Tedesco et al. (1), to 0.97 Zhou et al. (9)]. Jafari and colleagues achieved an accuracy of 0.96, while Mandalapu (19) and Zhu (3) similarly reported 0.96. Liu and colleagues reported 0.89, and Tedesco and colleagues (1) reported the lowest accuracy at 0.73, with the widest confidence interval reflecting their small sample size. The pooled fixed-effect estimate was 0.92 (95% CI: 0.89–0.95), indicating consistently high predictive accuracy across all wearable sensor studies. Heterogeneity was moderate (*I*² = 52.2%, *p* = 0.063), suggesting some variability between studies that did not reach statistical significance. These findings demonstrate that wearable sensor-based ML models achieve excellent pooled accuracy for RTS prediction, supporting their potential utility in clinical decision-making for athletes recovering from lower extremity injury, as shown in Figure 2 below. No markerless motion analysis studies reported accuracy for return-to-sport outcomes, representing a gap in the literature.

#### 4.1.2 Area under the receiver operating characteristic curve (AUC)

Only one wearable sensor study reported AUC for return-to-sport outcomes. Mandalapu et al. (19) achieved high discriminative performance for classifying left vs. right ACL-reconstructed knees using wearable IMU sensors with deep learning models. The CNN algorithm demonstrated AUCs of 0.95 for walking and 0.96 for jogging tasks (*N* = 89) at 3–6 months post-surgery. Notably, classification performance substantially declined when models were applied to longitudinal data at 9 months post-surgery (AUC dropped to 0.47–0.55), suggesting that movement patterns normalize over time as athletes recover. No other wearable sensor studies reported AUC values for RTS prediction. No markerless motion analysis studies reported AUC for return-to-sport outcomes. Given that only one study provided AUC data with limited longitudinal follow-up (*n* = 8 at visit 2), a pooled meta-analysis could not be performed. These findings highlight the need for standardized reporting of discriminative performance metrics in future RTS prediction research.

#### 4.1.3 Sensitivity

**Figure 3:**
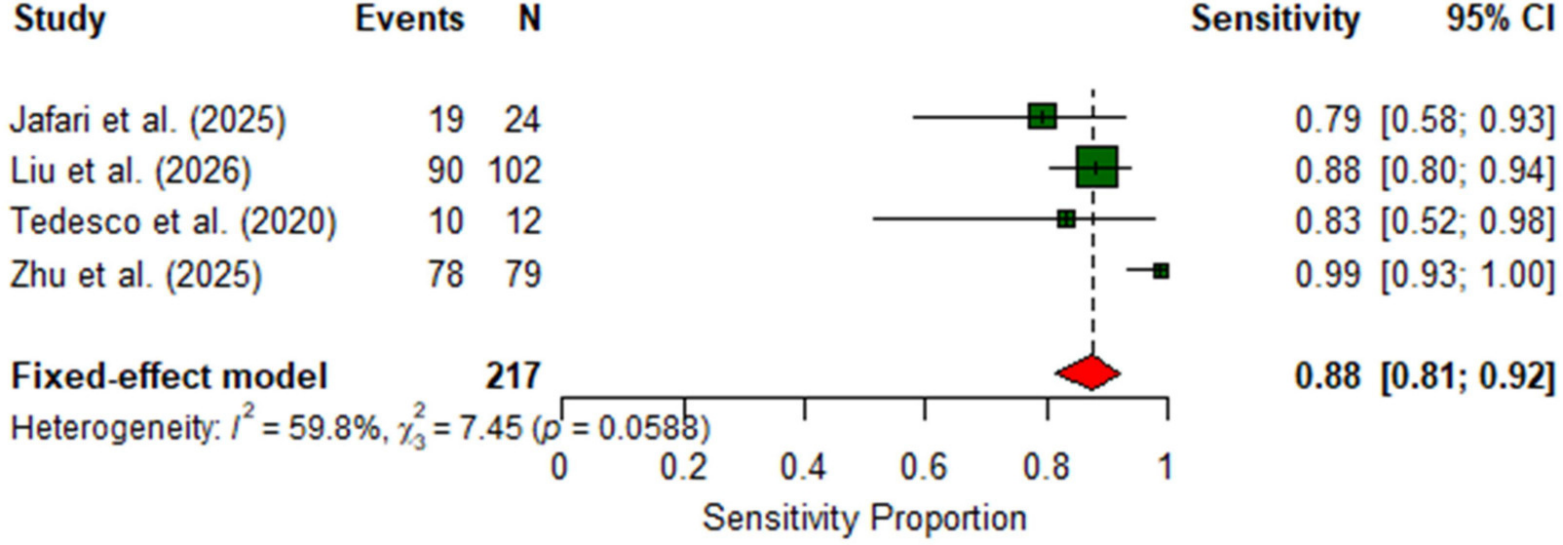
Sensitivity of wearable sensors (IMUs, EMG, or pressure insoles) for RTS.

Four studies employing wearable sensors reported sensitivity for return-to-sport classification following lower extremity injury (1, 3, 5, 8), as shown in Figure 3. No markerless studies reported sensitivity for RTS outcomes. Individual estimates ranged from 0.79 (8) to 0.99 (3). Zhu and colleagues achieved the highest sensitivity at 0.99 (95% CI: 0.93–1.00) using wearable IMU sensors with KNN classification. Liu and colleagues (5) reported good sensitivity of 0.88 (95% CI: 0.80–0.94) using a few-shot learning framework with pressure insoles and IMUs for chronic ankle instability assessment. Tedesco and colleagues reported 0.83 (95% CI: 0.52–0.98) using IMUs to discriminate post-ACL from healthy athletes, with the widest confidence interval reflecting their small sample size. Jafari and colleagues reported 0.79 (95% CI: 0.58–0.93) using sEMG signals and DCNN for ACL health level classification. The fixed-effect model showed moderate heterogeneity (*I*² = 59.8%, *p* = 0.059), indicating some variability across studies that did not reach statistical significance. These findings demonstrate that wearable sensor-based ML models achieve good to excellent sensitivity for RTS classification, correctly identifying injured athletes in the majority of cases. No markerless motion analysis studies reported sensitivity for return-to-sport outcomes.

#### 4.1.4 Specificity for RTS

Three studies employing wearable sensors reported specificity for classifying return-to-sport readiness following lower extremity injury (1, 3, 5). No markerless studies reported specificity for RTS outcomes. Values ranged from 0.745 to 0.99. Zhu and colleagues (3) (*N* = 79) achieved the highest specificity at 0.99 (95% CI: 0.96–1.00) using wearable IMU sensors and PSI-based features with KNN classification. Liu and colleagues (5) (*N* = 102) reported a good specificity of 0.89 (95% CI: 0.81–0.94) using a few-shot learning framework with pressure insoles and IMUs for chronic ankle instability rehabilitation assessment. Tedesco and colleagues (1) (*N* = 12) reported the lowest specificity at 0.745 (95% CI: 0.52–0.89) using IMUs and machine learning to discriminate post-ACL from healthy athletes. The small number of studies (K = 3) and substantial heterogeneity in sample sizes, injury types, and ML algorithms precluded meta-analytic pooling. Notably, the two larger studies demonstrated higher specificity (0.89 and 0.99) compared to the smallest study. These findings suggest that wearable sensor-based ML models can achieve excellent specificity for RTS classification, though validation in larger, homogenous cohorts is warranted. No markerless motion analysis studies reported specificity for return-to-sport outcomes.

#### 4.1.5 Precision and recall

No wearable sensor or markerless gait analysis study reported precision for return-to-sport Classification. Recall values were reported in two wearable sensor studies. Alzahrani et al. (4) reported a recall of 0.905 using a hybrid IMU-sEMG model with LSTM for injury-risk Classification. Zhou et al. (9) reported a recall of 0.667 using a Hybrid Transformer with markerless motion analysis. However, recall is conceptually identical to sensitivity, which has been reported separately. The absence of precision reporting across all studies is concerning because several RTS classification datasets were imbalanced, with sample sizes ranging from 12 to 102 participants. Without precision, the rate of false positive classifications—incorrectly clearing an athlete for return to sport—cannot be assessed. This represents a critical gap, as false positives in RTS decision-making could expose athletes to unnecessary re-injury risk. Given the scarcity of precision reporting and the redundancy of recall with sensitivity, these metrics were not suitable for meta-analytic pooling across studies.

### 4.2 Re-injury risk

#### 4.2.1 Accuracy

Two wearable sensor studies reported accuracy for re-injury risk prediction. Alzahrani et al. (4) achieved a high accuracy of 0.92 (95% CI: 0.81–0.97) using a hybrid IMU-sEMG model with LSTM. Xie (20) reported moderate accuracy of 0.83 (95% CI: 0.80–0.86) using ST-GNN with federated learning across multimodal wearable sensors (*N* = 500). One markerless study reported accuracy for re-injury risk prediction. Zhao et al. (13) reported the lowest accuracy among all re-injury studies at 0.66 (95% CI: 0.54–0.76) using markerless motion analysis and a Hybrid Transformer. Overall, accuracy ranged from 0.66 (markerless) to 0.92 (wearable). Due to the small number of studies (K = 3) and substantial heterogeneity in sample sizes, populations, ML models, and technologies, a pooled meta-analysis was not performed. Wearable sensors demonstrate promising accuracy for re-injury risk prediction, while markerless motion analysis requires further validation.

#### 4.2.2 Area under the receiver operating characteristic curve (AUC)

Two wearable sensor studies reported AUC for re-injury risk prediction (4, 13). Alzahrani and colleagues (4) (*N* = 50) achieved the highest AUC at 0.93 (95% CI: 0.84–0.97) using a hybrid IMU-sEMG model with bidirectional LSTM and attention mechanisms. Xie (20) (*N* = 500), the largest study, reported good discriminative ability with an AUC of 0.89 (95% CI: 0.84–0.92) using ST-GNN with federated meta-learning across multimodal wearable sensors. One markerless study reported an AUC for re-injury risk prediction. Zhao and colleagues (13) (*N* = 25) reported an AUC of 0.647 (95% CI: 0.49–0.77) using markerless motion analysis with a Hybrid Transformer, indicating poor discriminative performance approaching chance level. The small number of studies (K = 3) and substantial heterogeneity precluded meta-analytic pooling. Notably, wearable sensor studies (*N* ≥ 50) demonstrated good to excellent discriminative ability (AUC ≥ 0.89), while the single markerless study showed poor performance. These findings suggest that wearable sensor-based ML models can achieve high discriminative accuracy for re-injury risk, though larger validation studies are needed for markerless computer vision approaches.

#### 4.2.3 Sensitivity for re-injury risk

No wearable sensor study reported sensitivity for re-injury risk prediction. Two markerless studies reported sensitivity for re-injury risk prediction, precluding meta-analytic pooling. Hébert-Losier and colleagues (6) (*N* = 144) reported a sensitivity of 0.82 (95% CI: 0.75–0.87) using a random forest model applied to OpenPose-derived kinematic features from 2D video recordings for ACL injury risk screening via the Landing Error Scoring System. Zhao and colleagues (13) (*N* = 25) reported a lower sensitivity of 0.667 (95% CI: 0.52–0.79) using a Hybrid Transformer model with markerless 3D motion analysis. The wider confidence interval in the smaller study reflects greater estimation uncertainty. Substantial differences in sample size (144 vs. 25), injury populations, and ML approaches limit direct comparison. These findings highlight the scarcity of sensitivity reporting in re-injury risk prediction studies and underscore the need for standardized outcome reporting.

#### 4.2.4 Specificity for re-injury risk

No wearable sensor study reported specificity for re-injury risk prediction. Only one markerless study reported specificity for re-injury risk prediction. Hébert-Losier and colleagues (6) (*N* = 144) reported a specificity of 0.77 (95% CI: 0.68–0.84) using a random forest model applied to OpenPose-derived kinematic features from 2D video recordings. The model classified high vs. low ACL injury risk based on Landing Error Scoring System thresholds, achieving moderate specificity. This single estimate suggests that the model correctly identified low-risk individuals in approximately three-quarters of cases. The absence of specificity reporting across other re-injury risk prediction studies highlights a significant gap in the literature.

#### 4.2.5 Precision and recall

No study reported precision or recall for re-injury risk prediction following lower extremity injury. Alzahrani et al. (4) reported recall (0.905), but this study focused on injury-risk Classification rather than re-injury prediction specifically. Xie (20) mentioned F1-score in the evaluation section but did not report precision or recall values for the main model. The absence of precision and recall reporting across all included studies is particularly problematic because several studies had imbalanced datasets, where the injured class represented only 11 to 28 percent of the total sample. Without precision and recall, assessing how models performed on the minority injured class is impossible, and reported accuracy may be misleadingly inflated by majority class classification. The absence of precision and recall reporting across all included studies represents a significant gap, limiting the ability to assess model performance regarding false positives and false negatives.

### 4.3 Subgroup analyses

Subgroup analyses were not performed due to the limited number of studies reporting each predictive outcome. For return-to-sport readiness, accuracy was reported across six wearable sensor studies with moderate heterogeneity (*I*² = 52.2%), while specificity and sensitivity were reported in only three and four studies, respectively. For re-injury risk, accuracy, and AUC were each reported in only three studies, with sensitivity and specificity reported in two and one study, respectively. The small study size precluded meaningful subgroup comparisons across clinically relevant stratifiers such as injury type (ACL vs. ankle instability), ML algorithm category (traditional vs. deep learning), technology type (wearable sensors vs. markerless motion analysis), or risk of bias. Additionally, meta-regression to explore the influence of continuous moderators (e.g., sample size, participant age) was not feasible given the limited data points per outcome. Future updates of this review with a larger evidence base may enable subgroup analyses to investigate potential sources of heterogeneity, including differences in sensor placement, ML model architectures, validation methods, and follow-up durations.

### 4.4 Predictive performance across machine learning models

Across all included studies, ensemble and deep learning models generally outperformed traditional machine learning algorithms. Random forest achieved high specificity (0.94 to 0.99) and F1-scores (0.942 to 0.969) for adductor strain prediction (10), while gradient boosting demonstrated superior sensitivity (0.818) for discriminating post-ACLR from healthy athletes (1). Hybrid architectures combining IMU and EMG data with bidirectional LSTM and attention mechanisms achieved the highest overall accuracy (0.92) and AUC (0.93) for injury-risk classification (4). Convolutional neural networks performed well for feature fusion tasks, achieving 0.96 accuracy for CAI classification (9). K-nearest neighbors outperformed more complex models for ACL reconstruction outcome classification using PSI-based features, achieving 0.96 accuracy and 0.99 specificity (3). Transformers showed moderate performance (accuracy: 0.66, AUC: 0.647) but offered superior temporal interpretability through attention mechanisms (13). Few-shot learning improved accuracy by 0.17 compared to traditional models when training data were limited (5). These findings suggest that model selection should balance task-specific requirements, dataset size, and interpretability needs.

### 4.5 Biomechanical variables associated with return-to-sport outcomes

Several biomechanical variables emerged as consistent predictors across studies, with distinct patterns observed between wearable-derived and markerless-derived measurements. From wearable sensors, temporal gait parameters, including stride time, stance time, and swing time, were the most influential features for distinguishing ACL-injured from healthy individuals (21). Knee flexion angle and moment were repeatedly identified as critical predictors for both RTS readiness and re-injury risk, with reduced knee flexion associated with elevated injury susceptibility (22, 23). Muscle activation features, particularly quadriceps-hamstring ratios and EMG-derived synergy patterns, demonstrated strong discriminative capacity for CAI and ACLR populations (8, 9). Phase Slope Index causality features captured inter-limb coordination deficits persisting years post-surgery (3, 19). From markerless motion analysis, 2D video-derived kinematics via OpenPose achieved acceptable sensitivity and specificity for ACL injury risk screening (6). Asymmetry indices, including vertical ground reaction force asymmetry, were significant predictors of successful RTS (2). These findings support multifactorial assessment approaches combining kinematics, kinetics, and muscle activation patterns.

### 4.6 Validation approaches in prediction models

The choice of validation strategy fundamentally influenced reported predictive performance across all included studies. Participant-level cross-validation proved essential for preventing data leakage in repeated-measures designs, where multiple trials per participant create statistical dependencies (11). Leave-one-subject-out cross-validation produced conservative accuracy estimates ranging from 0.66 to 0.73, whereas standard k-fold cross-validation generated substantially higher estimates of 0.91. This dramatic difference occurred because standard k-fold cross-validation allowed trials from the same participant to appear in both training and test sets (11). The consequence of this data leakage is severe: models learned participant-specific patterns rather than generalisable injury-related biomechanics. Nested cross-validation frameworks demonstrated the best balance between generalisation and bias reduction by completely isolating hyperparameter tuning from final model evaluation. However, only one study implemented this rigorous approach, highlighting a critical methodological gap across the literature.

Most studies employed internal validation exclusively, using single datasets with train-test splits ranging from 70:30 to 80:20 (5, 8, 9). Only one study attempted external validation by applying a previously developed algorithm to an entirely new cohort. The results were striking: AUC dropped from 0.94 during internal validation to between 0.53 and 0.62 during external validation (2). This represents a performance degradation of approximately 35 percent, demonstrating that current models may not generalise across different populations, equipment, or clinical settings. SMOTE oversampling was used in small-sample studies to address class imbalance, improving F1-scores by 0.13 (10, 23). However, synthetic oversampling risks introducing artificial patterns not present in real data, potentially further reducing external validity. Bootstrapping for variable importance estimation was reported in some random forest models, but confidence intervals for performance metrics were inconsistently provided (10).

The complete absence of external validation across all 11 included studies represents the single most critical limitation of this evidence base. Without external validation in independent cohorts, reported accuracy, AUC, sensitivity, and specificity estimates cannot be trusted to generalise beyond the specific datasets on which models were trained. Few studies reported calibration metrics or decision curve analysis, limiting assessment of clinical utility and net benefit. Calibration—the agreement between predicted probabilities and observed outcome frequencies—is essential for clinical decision-making, yet was almost entirely absent. The combination of data leakage through inappropriate cross-validation, absence of external validation, and lack of calibration reporting means that current models are best characterised as exploratory rather than clinically deployable. Future research must prioritise prospective external validation in diverse independent cohorts, transparent reporting of calibration, and adherence to TRIPOD-AI guidelines before any model can be recommended for routine return-to-sport decision-making.

### 4.7 Clinical applicability and implementation feasibility of markerless systems

Markerless motion analysis systems offer distinct practical advantages over wearable sensors for return-to-sport assessment. However, the following conclusions are exploratory given the limited number of markerless studies (K = 3 for re-injury outcomes; zero for RTS accuracy), absence of external validation, and heterogeneity in populations and tasks. Smartphone-based pose estimation using OpenPose demonstrated acceptable sensitivity (0.82) and specificity (0.77) for ACL injury risk screening via the Landing Error Scoring System, enabling automated scoring without expert clinicians or depth cameras (6). OpenCap similarly provides three-dimensional kinematics from two iOS smartphones at less than one percent of traditional motion capture costs, though validation for RTS populations is ongoing (7). These systems eliminate the need for body-mounted sensors, reducing preparation time and addressing potential user discomfort or compliance issues. However, current markerless solutions have only been validated for injury risk screening in healthy populations, not for longitudinal RTS monitoring in injured athletes. Frame-level attention analysis in transformer models identified landing and push-off phases as most informative for injury classification, suggesting that targeted video segments may be sufficient for screening (13). Real-time feedback latency remains a challenge, with edge deployment on consumer smartphones requiring further optimization (24). Given the exploratory nature of these findings, the accessibility, low cost, and scalability of markerless systems remain promising but require prospective validation in injured RTS populations.

Several barriers must be addressed before markerless systems achieve widespread clinical adoption, and all interpretations remain exploratory due to the small evidence base. Accuracy for frontal and transverse plane kinematics remains reduced compared to sagittal plane measures, particularly for hip rotation and knee valgus assessment (13). Markerless systems also demonstrated poorer performance for re-injury risk prediction (AUC: 0.647) compared to wearable sensors (AUC: 0.89–0.93) (4, 13, 20). Lighting conditions, camera angles, and occlusions affect pose estimation quality in uncontrolled environments (6). Current implementations require specific camera placements and calibration procedures, limiting true field deployment (7). Critically, no markerless study reported accuracy for return-to-sport outcomes specifically, representing a major evidence gap. Computational requirements for deep learning-based pose estimation currently exceed consumer smartphone capabilities, though model compression techniques are advancing rapidly (6). Privacy concerns related to video data transmission require edge-based processing solutions (24). Future development should prioritize real-time feedback optimization, external validation across diverse clinical populations, and integration with existing rehabilitation workflows. These exploratory findings underscore the need for larger, prospective markerless studies before clinical implementation can be recommended.

### 4.8 Influence of injury type

The influence of injury type on predictive performance could not be systematically evaluated due to heterogeneity in outcome reporting and limited study numbers per injury category. ACL injury was the most frequently studied condition, comprising four wearable sensor studies for RTS prediction (1, 3, 8, 19) and one markerless study for re-injury risk (13). Chronic ankle instability was addressed in two wearable sensor studies, both reporting excellent accuracy (0.89–0.96) for rehabilitation status classification (5, 9). Adductor strain prediction was limited to one retrospective cohort study using wearable GPS/IMU data, with F1-scores ranging from 0.932 to 0.969 (10). This substantial heterogeneity across injury types has direct implications for meta-analytic pooling: combining ACL, CAI, and adductor injury studies into a single pooled estimate assumes that predictive performance is comparable across mechanically distinct conditions. This assumption is almost certainly violated, as ACL involves ligamentous instability, CAI involves sensorimotor deficits, and adductor strains involve muscle-tendon unit failure. Sensitivity and specificity varied substantially across injury types, with ACL studies reporting broader ranges (sensitivity: 0.79–0.99; specificity: 0.75–0.99) (1, 3, 8) compared to CAI studies (sensitivity: 0.88; specificity: 0.89) (5). Larger, injury-specific cohorts are needed to determine whether prediction models generalise across different lower extremity conditions or require condition-specific algorithm development.

### 4.9 GRADE quality evaluation

The quality of evidence across outcomes was assessed using the GRADE framework, considering study design, risk of bias, inconsistency, indirectness, imprecision, and publication bias. Most evidence originated from cross-sectional prediction model development studies with small sample sizes and internal validation only. Consequently, the quality of evidence ranged from moderate to very low across all outcomes. Downgrading decisions primarily reflected serious risk of bias due to the absence of external validation, substantial heterogeneity across studies, imprecision from wide confidence intervals, and indirectness of outcome measures to clinically meaningful RTS endpoints.

## 5 Discussion

### 5.1 Interpreting predictive accuracy in a clinical context

The pooled accuracy for return-to-sport classification using wearable sensors was 0.92 (95% CI: 0.89–0.95), suggesting that current machine learning models achieve excellent discriminative performance (1, 3–5, 8, 9, 19). However, clinicians must interpret this pooled estimate cautiously. First, accuracy alone does not reflect clinical utility; sensitivity (0.79–0.99) and specificity (0.75–0.99) varied considerably across studies (1, 3, 8). Moreover, most studies employed internal validation only, and external validation attempts showed substantial performance drops Richter et al. (2). Moreover, re-injury risk prediction accuracy was lower (0.66–0.92) with wider variability (4, 13, 20). Predictive accuracy also depends on injury type, task, and population. For instance, chronic ankle instability classification achieved higher accuracy (0.96) than ACL re-injury prediction (AUC: 0.647) (9, 13). Clinicians should therefore view these accuracy estimates as promising but not definitive. Real-world performance will likely be lower than reported due to differences in patient populations, testing environments, and the absence of standardized RTS criteria. Prediction models require external validation before clinical implementation (2).

### 5.2 The validation gap and its implications for clinical trust

A critical finding of this review is the near-total absence of external validation across included studies. Only one study attempted external validation by applying a previously developed algorithm to a new cohort, resulting in dramatic performance degradation (AUC dropping from 0.94 to 0.53–0.62) (2). This validation gap severely limits clinical trust and generalizability. Internal validation techniques varied widely: leave-one-subject-out cross-validation produced conservative accuracy estimates (0.66–0.73), while standard k-fold cross-validation inflated performance (0.91) due to data leakage (11). Nested cross-validation frameworks demonstrated better generalization but were rarely implemented (11). SMOTE oversampling improved F1-scores by 0.13 in small-sample studies but may introduce synthetic data artifacts (10, 23). Without rigorous external validation in independent cohorts, current models risk being overfitted to specific datasets, limiting their applicability across different clinical settings, populations, and equipment types (11). Future research must prioritize multi-centre prospective validation and transparent reporting of calibration metrics. Collaborative data-sharing initiatives would enable more robust external validation across diverse athletic populations.

### 5.3 Algorithm complexity versus clinical accessibility

The most accurate models in this review were also the most computationally complex. Hybrid architectures combining IMU and EMG data with bidirectional LSTM and attention mechanisms achieved the highest accuracy (0.92) and AUC (0.93) but require substantial computational resources and expert oversight (4). Conversely, simpler models like K-nearest neighbors achieved comparable performance (accuracy: 0.96) for PSI-based feature classification with lower computational demands (3). This trade-off between algorithm complexity and clinical accessibility is critical for real-world deployment. Markerless systems using smartphone-based pose estimation reduce hardware costs but currently show poorer discriminative performance for re-injury risk (AUC: 0.647) compared to wearable sensors (6, 13). Furthermore, edge deployment of deep learning models on consumer smartphones remains challenging due to latency and computational constraints (24, 25). Clinicians and sports practitioners must therefore balance predictive accuracy against practical feasibility. For resource-limited settings, simpler validated algorithms may offer more sustainable solutions than complex black-box models requiring specialized infrastructure (1, 19).

### 5.4 The neglected role of psychosocial factors

A striking omission across all included studies is the absence of psychosocial variables in prediction models. Fear of re-injury, kinesiophobia, and psychological readiness are well-established predictors of return-to-sport outcomes following lower extremity injury. One study explicitly classified fear of re-injury using biomechanical features, demonstrating that movement patterns alone can capture psychological states (11). However, no study integrated patient-reported outcome measures of psychological readiness directly into predictive algorithms alongside biomechanical data. This represents a missed opportunity, as psychological factors mediate the relationship between physical recovery and successful RTS. The biomechanical features identified as important predictors—such as reduced knee flexion, muscle activation asymmetries, and altered coordination patterns—may themselves be manifestations of underlying fear or low confidence (2, 11). Future prediction models should adopt a biopsychosocial framework, integrating patient-reported psychological measures, clinical examination findings, and wearable sensor data. This holistic approach would more accurately reflect the multifactorial nature of RTS readiness (2, 3). Without such integration, models risk oversimplifying complex recovery trajectories.

### 5.5 Wearable technology and equity of access to biomechanical assessment

The cost and accessibility of biomechanical assessment technologies raise important equity considerations. Markerless systems using smartphone cameras (e.g., OpenPose, OpenCap) cost less than one percent of traditional motion capture systems and eliminate the need for expensive wearable sensors (6, 7). This democratization of movement analysis could enable large-scale injury risk screening in community sports settings where laboratory access is limited (26). However, current markerless solutions have not been validated for return-to-sport outcomes in injured populations, and their accuracy for frontal and transverse plane kinematics remains reduced compared to sagittal plane measures (13). Conversely, wearable sensors (IMUs, EMG, pressure insoles) achieve higher predictive accuracy but require equipment purchase (approximately 175–1,000 + USD), sensor attachment, and technical expertise for data interpretation (1, 5). This creates a two-tier system: well-resourced sports medicine facilities may access high-accuracy wearable systems, while community and amateur settings rely on less-validated markerless alternatives. Without targeted implementation research and funding models, these technologies risk widening existing disparities in sports medicine access (25, 27).

### 5.6 Implications for elite sport performance and injury surveillance

For elite sport settings, wearable sensors combined with machine learning offer actionable opportunities for longitudinal injury surveillance. The high accuracy (0.92) and AUC (0.89–0.93) achieved by hybrid IMU-EMG models suggest that these systems could be integrated into routine athlete monitoring protocols (4, 20). Real-time feedback latency of 188 milliseconds enables immediate technique correction during training (4). However, several barriers limit current adoption. First, the absence of external validation means models may not generalize across different teams, sports, or equipment types (2). Also, most studies used treadmill-based protocols, which may not fully replicate overground sport-specific movements (25, 28). Moreover, the heterogeneity in ML architectures and feature sets prevents standardization across organizations. Fourth, data privacy concerns regarding biometric information require a secure infrastructure (24). Elite teams should therefore view current models as supplementary decision-support tools rather than definitive RTS arbiters. Collaborative efforts between sports scientists, clinicians, and data scientists are needed to establish validation protocols and share anonymized datasets. Prospective implementation studies tracking injury outcomes over full competitive seasons are urgently required (29).

### 5.7 Research policy and the need for reporting standardisation

This review identifies critical gaps in reporting transparency that impede meta-analytic synthesis and clinical translation. Only three studies reported AUC for re-injury risk (4, 13, 20), and specificity was reported in only three studies for RTS outcomes (1, 3, 5). Precision and recall were almost absent. Crucially, no markerless study reported accuracy for return-to-sport outcomes specifically (6, 7, 13). This inconsistent reporting prevents meaningful comparisons across studies and limits the ability to assess model performance comprehensively. Key missing elements included calibration metrics, decision curve analysis, handling of missing data, and specification of the intended clinical application context. Furthermore, few studies reported confidence intervals for performance metrics or conducted sensitivity analyses for key assumptions (30). Research funding bodies and journals should mandate adherence to established reporting standards as a condition of publication. Collaborative data-sharing initiatives would also enable external validation and meta-analytic pooling, accelerating progress toward clinically deployable prediction models for return-to-sport decision-making (31).

### 5.8 Limitations

This review provides a structured synthesis of current evidence but also includes several limitations that affect interpretation. Substantial heterogeneity exists across the included studies in terms of design, populations, and outcome measures. Differences in model types and data sources limit comparability and reduce the precision of pooled estimates. Low intraclass correlation coefficients observed for temporal gait parameters, including stance time and step time, warrant clinical consideration. These lower ICC values indicate greater between-session variability in timing-related biomechanical measures compared to kinematic parameters such as knee flexion angle. Clinicians should prioritise kinematic and kinetic parameters over temporal parameters for return-to-sport decisions, as the latter demonstrate less consistent reliability across repeated assessments.

Many studies rely on small datasets and internal validation, increasing the risk of overfitting and bias. Cross-sectional designs limit causal inference and restrict understanding of long-term outcomes. Inconsistent reporting further reduces confidence in findings and complicates synthesis. The generalizability of findings to populations beyond those represented is limited. Most included studies focused on ACL injury and chronic ankle instability, with limited representation of knee osteoarthritis, patellofemoral pain, Achilles tendinopathy, or stress fractures. The predominance of young adult athletic populations restricts applicability to older adults, sedentary individuals, or those with complex multimorbidities. Validation across diverse injury types, age groups, and activity levels is required before clinical implementation can be recommended.

### 5.9 Future research

Future research must prioritize multi-centre external validation as the single most critical gap identified in this review. No included study conducted external validation in an independent cohort, and the one study that attempted cross-validation on a different sample showed substantial performance decline (2). Researchers should therefore prioritize prospective validation of existing algorithms in diverse independent populations before claiming clinical utility. Multi-centre collaborations are essential to establish whether models generalize across different sports, injury types, rehabilitation protocols, and wearable device configurations. Also, standardized outcome reporting is urgently needed. Future studies should consistently report sensitivity, specificity, precision, recall, F1-score, AUC, and calibration metrics alongside accuracy, with confidence intervals for all estimates. Adherence to TRIPOD-AI and PROBAST guidelines would facilitate meta-analytic synthesis. Third, longitudinal prospective studies tracking actual RTS success and re-injury events over 12–24 months are required, as most current studies are cross-sectional or use surrogate biomechanical endpoints. Fourth, markerless systems require dedicated validation for RTS outcomes, as current evidence is limited to injury risk screening in healthy populations. Fifth, integration of psychosocial factors (fear of re-injury, psychological readiness) with biomechanical data into predictive models is needed. Finally, implementation research examining real-world feasibility, clinician adoption, and cost-effectiveness is essential before widespread deployment.

## 6 Conclusion

This systematic review and meta-analysis evaluated the predictive accuracy of machine learning combined with markerless gait analysis and wearable sensors for return-to-sport following lower extremity injury. Wearable sensor-based models achieved excellent pooled accuracy for RTS classification (0.92, 95% CI: 0.89–0.95) and good to excellent discriminative ability for re-injury risk (AUC: 0.89–0.93). Markerless motion analysis demonstrated acceptable sensitivity (0.82) and specificity (0.77) for injury risk screening but showed poor performance for re-injury prediction (AUC: 0.647) and no evidence for RTS outcomes. However, the quality of evidence ranged from low to very low across all outcomes. Critically, no study conducted external validation in an independent cohort, and substantial heterogeneity, small sample sizes, and inconsistent outcome reporting limited meta-analytic synthesis. Precision, recall, and calibration metrics were rarely reported. These findings demonstrate that wearable sensor-based machine learning models hold promise for RTS decision support, but current evidence remains exploratory without prospective validation in injured athletic populations. Markerless systems require dedicated validation for RTS outcomes before clinical implementation. Future research must prioritise multi-centre external validation, standardised outcome reporting, and integration of psychosocial factors to advance these technologies from research settings into evidence-based clinical practice.

## Data Availability

The original contributions presented in the study are included in the article/Supplementary Material, further inquiries can be directed to the corresponding author.
