# Supplementary material for: Predictive accuracy of machine learning and markerless gait analysis for return-to-sport following lower extremity injury: a systematic review and meta-analysis
Source: Front Sports Act Living. 2026 Jun 16;8:1862870. doi: 10.3389/fspor.2026.1862870 (PMC13314457; doi:10.3389/fspor.2026.1862870)
Supplement: Supplementary file 1 [file Supplementaryfile1.pdf]

Appendix

Table 2: Study Characteristics

Author (Year)	Country	Design	N	Outcome Type (RTS/Re-Injury)	Injury Type	ML Model Type	Technology Type	Validation Type
**Alzahrani et al. (2026)**	Pakistan	Cross-sectional / prediction model	50	Re-injury risk	ACL and muscle-strain risk	Bi-LSTM hybrid model	Wearable sensors (IMU + sEMG)	Internal (10-fold CV)
**Davis et al. (2026)**	USA	Retrospective cohort	90	Re-injury risk	Adductor strain	Random Forest (RF) with SMOTE	Wearable sensors + strength testing	Internal (80/20 split, bootstrapping)
**Hébert-Losier et al. (2020)**	New Zealand	Cross-sectional (technical validation)	144	Re-injury risk	ACL injury risk	Random Forest (regression)	Markerless computer vision (OpenPose)	Internal (10×10-fold CV)
**Jafari et al. (2025)**	Iran	Cross-sectional / prediction model	24	RTS rehabilitation status	Post-ACLR	DCNN	Wearable sensors (sEMG)	Internal (80/20 split)
**Liu et al. (2026)**	China	Cross-sectional / prediction model	102	RTS rehabilitation status	Chronic lateral ankle instability	Few-shot learning (MLP/CNN/LSTM)	Wearable sensors (pressure insoles + IMUs)	Internal (80/20 split)
**Mandalapu et al. (2021)**	USA	Longitudinal	89	RTS rehabilitation status	Post-ACLR	CNN, LSTM, GRU, RNN	Wearable sensors (5 IMUs)	Internal (5-fold CV)
**Tedesco et al. (2020)**	Ireland	Cross-sectional	12	RTS rehabilitation status	Post-ACLR	kNN, NB, SVM, XGB, MLP, Stacking	Wearable sensors (IMUs)	Internal (LOSO-CV)
**Zhao et al. (2026)**	Canada	Prospective cohort	25	Re-injury risk	Non-contact lower limb injuries	SVM, XGBoost, LSTM, Hybrid Transformer	Markerless 3D motion analysis	Internal (LOSO-CV)

**Zhou et al. (2026)**	China	Cross-sectional	60	RTS rehabilitation status	Chronic ankle instability	CNN + Random Forest	Wearable sensors (sEMG)	Internal (70/30 split, OOB)
**Zhu et al. (2025)**	USA	Cross-sectional	79	RTS rehabilitation status	Post-ACLR	KNN, SVM, NB, RF, NN	Wearable sensors (5 IMUs)	Internal (5-fold CV)
**Xie (2025)**	China	Cross-sectional / prediction model	500	Re-injury risk	ACL injury	ST-GNN + federated learning	Wearable sensors (IMU + sEMG + PPG)	Internal (70/20/10 split)

Table 3: Search Results by Database

Data Base	Terms	Results
**PubMed**	("Machine Learning"[Mesh] OR "Artificial Intelligence"[Mesh] OR machine learning OR deep learning OR artificial intelligence OR AI OR predictive model* OR algorithm* OR neural network*)<br>AND<br>("Gait"[Mesh] OR "Gait Analysis"[Mesh] OR gait OR biomechanics OR "motion analysis" OR "markerless motion capture" OR "markerless gait analysis" OR video-based analysis)<br>AND<br>("Return to Sport"[Mesh] OR "return to sport" OR RTS OR "return to play" OR reinjur* OR "injury risk" OR prognos*)<br>AND<br>("Lower Extremity"[Mesh] OR "Athletic Injuries"[Mesh] OR "running injury" OR runner* OR "ACL" OR "anterior cruciate ligament" OR "Achilles tendinopathy" OR "hamstring injury" OR "patellofemoral pain" OR "stress fracture*" OR "ankle sprain*")	227
**Embase**	(("Machine Learning" or "Artificial Intelligence" or machine learning or deep learning or artificial intelligence or AI or predictive model or algorithm or neural network) and ("Gait" or "Gait Analysis" or gait or biomechanics or "motion analysis" or "markerless motion capture" or "markerless gait analysis" or video-based analysis) and ("Return to Sport" or "return to sport" or RTS or "return to play" or reinjur or "injury risk" or prognos) and ("Lower Extremity" or "Athletic Injuries" or "running injur" or runner or "ACL" or "anterior cruciate ligament" or "Achilles tendinopathy" or "hamstring injury" or "patellofemoral pain" or "stress fracture" or "ankle sprain")).mp. [mp=title, abstract, heading word, drug trade name, original title, device manufacturer, drug manufacturer, device trade name, keyword heading word, floating subheading word, candidate term word]	74
**Scopus**	TITLE-ABS-KEY<br>("machine learning" OR "artificial intelligence" OR "deep learning" OR<br>"neural network*" OR "computer vision" OR "predictive model*" OR algorithm*)<br>AND<br>TITLE-ABS-KEY<br>(gait OR "gait analysis" OR biomechanics OR "motion analysis" OR<br>"movement analysis" OR "human motion" OR "pose estimation" OR<br>"markerless" OR "video analysis")<br>AND<br>TITLE-ABS-KEY<br>("return to sport" OR "return to play" OR RTS OR reinjur* OR<br>"injury risk" OR predict* OR prognos*)<br>AND<br>TITLE-ABS-KEY<br>("lower extremity" OR "lower limb" OR running OR runner* OR<br>"sports injury" OR musculoskeletal OR knee OR ankle OR hip)	1, 434<br>Filter:<br>Medicine<br>Biology
**IEEE Xplore**	(MH "Machine Learning" OR MH "Artificial Intelligence" OR machine learning OR artificial intelligence OR deep learning OR neural network* OR predictive model*)<br>AND<br>(MH "Gait" OR MH "Gait Analysis" OR gait OR biomechanics OR "motion analysis" OR "markerless motion capture")<br>AND<br>(MH "Return to Sport" OR "return to sport" OR "return to play" OR RTS OR reinjur* OR "injury risk")<br>AND<br>(MH "Lower Extremity" OR MH "Athletic Injuries" OR "running injur*" OR runner* OR ACL OR "Achilles tendinopathy" OR "hamstring injury" OR "patellofemoral pain" OR "stress fracture*" OR "ankle sprain*")	15

**CINAHL**	(MH "Machine Learning" OR MH "Artificial Intelligence" OR machine learning OR artificial intelligence OR deep learning OR neural network* OR predictive model*) AND (MH "Gait" OR MH "Gait Analysis" OR gait OR biomechanics OR "motion analysis" OR "markerless motion capture") AND (MH "Return to Sport" OR "return to sport" OR "return to play" OR RTS OR reinjur* OR "injury risk") AND (MH "Lower Extremity" OR MH "Athletic Injuries" OR "running injur*" OR runner* OR ACL OR "Achilles tendinopathy" OR "hamstring injury" OR "patellofemoral pain" OR "stress fracture*" OR "ankle sprain*")	12

Table 4: PROBAST Risk of Bias and Applicability Assessment

Study ID	Participants (ROB)	Predictors (ROB)	Outcome (ROB)	Analysis (ROB)	Overall ROB	Participants (Applicability)	Predictors (Applicability)	Outcome (Applicability)	Overall Applicability
1. Alzahrani et al. (2026)	Low	Low	Low	High	High	Low	Low	Low	Low
2. Davis et al. (2026)	Low	Low	Low	High	High	Low	Low	Low	Low
3. Hébert-Losier et al. (2020)	Low	Low	Low	High	High	Low	Low	Low	Low
4. Jafari et al. (2025)	Low	Low	Low	High	High	Low	Low	Low	Low
5. Liu et al. (2026)	Low	Low	Low	High	High	Low	Low	Low	Low
6. Mandalapu et al. (2021)	Low	Low	Low	High	High	Low	Low	Low	Low
7. Tedesco et al. (2020)	Low	Low	Low	High	High	Low	Low	Low	Low
8. Zhao et al. (2026)	Low	Low	Low	High	High	Low	Low	Low	Low
9. Zhou et al. (2026)	Low	Low	Low	High	High	Low	Low	Low	Low
10. Zhu et al. (2025)	Low	Low	Low	High	High	Low	Low	Low	Low

**11. Xie (2025)**	Low	Low	Low	High	High	Low	Low	Low	Low
Overall (most frequent)	**Low**	**Low**	**Low**	**High**	**High**	**Low**	**Low**	**Low**	**Low**

RTS – Accuracy

Study ID	Sample Size (N)	Accuracy (%)	Accuracy (p)	logit(p)	Variance	95% CI for logit	95% CI for Accuracy
**Jafari et al. (2025)**	24	90.2–94.0%	0.902	2.220	0.210	(1.322, 3.118)	(0.790, 0.958)
**Liu et al. (2026)**	102	89%	0.890	2.090	0.100	(1.470, 2.710)	(0.813, 0.938)
**Mandalapu et al. (2021)**	89	95.16–96.40%	0.952	2.988	0.050	(2.133, 3.843)	(0.894, 0.979)
**Tedesco et al. (2020)**	12	73.07%	0.731	0.999	0.439	(0.022, 1.976)	(0.506, 0.878)
**Zhou et al. (2026)**	60	96%	0.960	3.178	0.060	(2.503, 3.853)	(0.924, 0.979)
**Zhu et al. (2025)**	79	93–96.37%	0.930	2.586	0.069	(1.997, 3.175)	(0.880, 0.960)

Pooled Fixed-Effect Estimate: 0.92 (95% CI: 0.89–0.95) | **Heterogeneity:** $I^2 = 52.2\%$, $p = 0.063$

RTS – AUC

Study ID	AUC	SE	logit(AUC)	Variance	95% CI for logit	95% CI for AUC
**Jafari et al. (2025)**	NR	—	—	—	—	—
**Liu et al. (2026)**	NR	—	—	—	—	—
**Mandalapu et al. (2021)**	0.95–0.96	0.022	2.944	0.036	(2.433, 3.455)	(0.920, 0.969)
**Tedesco et al. (2020)**	NR	—	—	—	—	—
**Zhou et al. (2026)**	NR	—	—	—	—	—
**Zhu et al. (2025)**	NR	—	—	—	—	—

Note: Pooled meta-analysis not performed (only one study reported AUC).

RTS – Sensitivity

Study ID	N	Sensitivity (%)	Sensitivity (p)	logit(p)	Variance	95% CI for logit	95% CI for Sensitivity
**Jafari et al. (2025)**	24	78–96%	0.790	1.324	0.218	(0.508, 2.140)	(0.624, 0.895)
**Liu et al. (2026)**	102	88%	0.880	1.992	0.098	(1.470, 2.514)	(0.813, 0.925)
**Tedesco et al. (2020)**	12	81.8%	0.818	1.503	0.345	(0.250, 2.756)	(0.562, 0.940)
**Zhu et al. (2025)**	79	95–99%	0.990	4.595	0.256	(3.179, 6.011)	(0.960, 0.998)

Heterogeneity: $I^2 = 59.8\%$, $p = 0.059$ (pooled meta-analysis not performed due to heterogeneity)

RTS – Specificity

Study ID	N	Specificity (%)	Specificity (p)	logit(p)	Variance	95% CI for logit	95% CI for Specificity
**Jafari et al. (2025)**	24	NR	—	—	—	—	—
**Liu et al. (2026)**	102	89%	0.890	2.090	0.100	(1.470, 2.710)	(0.813, 0.938)
**Mandalapu et al. (2021)**	89	NR	—	—	—	—	—
**Tedesco et al. (2020)**	12	74.5%	0.745	1.072	0.439	(0.095, 2.049)	(0.524, 0.886)
**Zhou et al. (2026)**	60	NR	—	—	—	—	—
**Zhu et al. (2025)**	79	97–99%	0.990	4.595	0.256	(3.179, 6.011)	(0.960, 0.998)

Note: Pooled meta-analysis not performed (K=3 studies only, substantial heterogeneity).

Re-injury Risk – Accuracy

Study ID	N	Accuracy (%)	Accuracy (p)	logit(p)	Variance	95% CI for logit	95% CI for Accuracy
**Alzahrani et al. (2026)**	50	92.3%	0.923	2.483	0.281	(1.440, 3.526)	(0.808, 0.971)
**Davis et al. (2026)**	90	NR	—	—	—	—	—
**Hébert-Losier et al. (2020)**	144	NR	—	—	—	—	—
**Zhao et al. (2026)**	25	66.0%	0.660	0.663	0.178	(0.169, 1.157)	(0.542, 0.761)
**Xie (2025)**	500	83%	0.830	1.585	0.014	(1.353, 1.817)	(0.795, 0.860)

Note: Pooled meta-analysis not performed (K=3 studies only, substantial heterogeneity)

Re-injury Risk – AUC

Study ID	N	AUC	SE	logit(AUC)	Variance	95% CI for logit	95% CI for AUC
**Alzahrani et al. (2026)**	50	0.93	0.035	1.815	0.307	(0.720, 2.910)	(0.836, 0.974)
**Davis et al. (2026)**	90	NR	—	—	—	—	—
**Hébert-Losier et al. (2020)**	144	NR	—	—	—	—	—
**Zhao et al. (2026)**	25	0.647	0.061	-0.877	0.175	(-1.697, -0.057)	(0.490, 0.767)
**Xie (2025)**	500	0.89	0.018	1.265	0.020	(0.988, 1.542)	(0.844, 0.920)

Note: Pooled meta-analysis not performed (K=3 studies only, substantial heterogeneity)

Re-injury Risk – Sensitivity

Study ID	N	Sensitivity (%)	Sensitivity (p)	logit(p)	Variance	95% CI for logit	95% CI for Sensitivity
**Alzahrani et al. (2026)**	50	NR	—	—	—	—	—
**Davis et al. (2026)**	90	NR	—	—	—	—	—
**Hébert-Losier et al. (2020)**	144	82%	0.820	1.516	0.047	(1.111, 1.921)	(0.752, 0.872)
**Zhao et al. (2026)**	25	66.7%	0.667	0.695	0.180	(0.091, 1.299)	(0.523, 0.786)
**Xie (2025)**	500	NR	—	—	—	—	—

Note: Pooled meta-analysis not performed (K=2 studies only)

Re-injury Risk – Specificity

Study ID	N	Specificity (%)	Specificity (p)	logit(p)	Variance	95% CI for logit	95% CI for Specificity
**Alzahrani et al. (2026)**	50	NR	—	—	—	—	—
**Davis et al. (2026)**	90	NR	—	—	—	—	—
**Hébert-Losier et al. (2020)**	144	77%	0.770	1.208	0.039	(0.759, 1.657)	(0.681, 0.840)

**Zhao et al. (2026)**	25	NR	—	—	—	—	—
**Xie (2025)**	500	NR	—	—	—	—	—

Note: Only one study reported specificity; pooled meta-analysis not performed.

Supplementary Search Strings

Data Base	Terms	Results
**PubMed**	("Machine Learning"[Mesh] OR "Artificial Intelligence"[Mesh] OR machine learning OR deep learning OR artificial intelligence OR AI OR predictive model* OR algorithm* OR neural network*)<br>AND<br>("Gait"[Mesh] OR "Gait Analysis"[Mesh] OR gait OR biomechanics OR "motion analysis" OR "markerless motion capture" OR "markerless gait analysis" OR video-based analysis)<br>AND<br>("Return to Sport"[Mesh] OR "return to sport" OR RTS OR "return to play" OR reinjur* OR "injury risk" OR prognos*)<br>AND<br>("Lower Extremity"[Mesh] OR "Athletic Injuries"[Mesh] OR "running injur*" OR runner* OR "ACL" OR "anterior cruciate ligament" OR "Achilles tendinopathy" OR "hamstring injury" OR "patellofemoral pain" OR "stress fracture*" OR "ankle sprain*")	227
**Embase**	(("Machine Learning" or "Artificial Intelligence" or machine learning or deep learning or artificial intelligence or AI or predictive model or algorithm or neural network) and ("Gait" or "Gait Analysis" or gait or biomechanics or "motion analysis" or "markerless motion capture" or "markerless gait analysis" or video-based analysis) and ("Return to Sport" or "return to sport" or RTS or "return to play" or reinjur or "injury risk" or prognos) and ("Lower Extremity" or "Athletic Injuries" or "running injur" or runner or "ACL" or "anterior cruciate ligament" or "Achilles tendinopathy" or "hamstring injury" or "patellofemoral pain" or "stress fracture" or "ankle sprain")).mp. [mp=title, abstract, heading word, drug trade name, original title, device manufacturer, drug manufacturer, device trade name, keyword heading word, floating subheading word, candidate term word]	74
**Scopus**	TITLE-ABS-KEY<br>("machine learning" OR "artificial intelligence" OR "deep learning" OR "neural network*" OR "computer vision" OR "predictive model*" OR algorithm*)<br>AND<br>TITLE-ABS-KEY<br>(gait OR "gait analysis" OR biomechanics OR "motion analysis" OR "movement analysis" OR "human motion" OR "pose estimation" OR "markerless" OR "video analysis")	1, 434<br>Filter:<br>Medicine<br>Biology

	AND<br>TITLE-ABS-KEY<br>("return to sport" OR "return to play" OR RTS OR reinjur* OR<br>"injury risk" OR predict* OR prognos*)<br>AND<br>TITLE-ABS-KEY<br>("lower extremity" OR "lower limb" OR running OR runner* OR<br>"sports injury" OR musculoskeletal OR knee OR ankle OR hip)	
**IEEE Xplore**	(MH "Machine Learning" OR MH "Artificial Intelligence" OR machine learning OR artificial intelligence OR deep learning OR neural network* OR predictive model*)<br>AND<br>(MH "Gait" OR MH "Gait Analysis" OR gait OR biomechanics OR "motion analysis" OR "markerless motion capture")<br>AND<br>(MH "Return to Sport" OR "return to sport" OR "return to play" OR RTS OR reinjur* OR "injury risk")<br>AND<br>(MH "Lower Extremity" OR MH "Athletic Injuries" OR "running injur*" OR runner* OR ACL OR "Achilles tendinopathy" OR "hamstring injury" OR "patellofemoral pain" OR "stress fracture*" OR "ankle sprain*")	15
**CINAHL**	(MH "Machine Learning" OR MH "Artificial Intelligence" OR machine learning OR artificial intelligence OR deep learning OR neural network* OR predictive model*) AND (MH "Gait" OR MH "Gait Analysis" OR gait OR biomechanics OR "motion analysis" OR "markerless motion capture") AND (MH "Return to Sport" OR "return to sport" OR "return to play" OR RTS OR reinjur* OR "injury risk") AND (MH "Lower Extremity" OR MH "Athletic Injuries" OR "running injur*" OR runner* OR ACL OR "Achilles tendinopathy" OR "hamstring injury" OR "patellofemoral pain" OR "stress fracture*" OR "ankle sprain*")	12
